# Short and Long-term Nursing Home Use in Older Medicaid-funded Home and Community-based Service Recipients

**DOI:** 10.1093/geroni/igaf122.400

**Published:** 2025-12-31

**Authors:** Richard Fortinsky, Julie Robison, James Grady, David Steffens, Chae Man Lee, Kristin Baker, Deborah Migneault

**Affiliations:** University of Connecticut, Farmington, Connecticut, United States; University of Connecticut, Farmington, Connecticut, United States; University of Connecticut School of Medicine, Farmington, Connecticut, United States; University of Connecticut, Farmington, Connecticut, United States; University of Connecticut, Farmington, Connecticut, United States; University of Connecticut, Farmington, Connecticut, United States; University of Connecticut, Farmington, Connecticut, United States

## Abstract

Medicaid-funded home and community-based service (HCBS) programs for older adults are receiving increasing attention from policymakers and researchers. While HCBS programs are intended to delay or avoid nursing home (NH) admissions, little is known about patterns of short-term and long-term NH admissions, or about factors that confer greater risk for NH admission, among HCBS recipients. We determined predictors of both types of NH admissions in a statewide population of HCBS recipients aged >65, classifying predictors as predisposing, enabling, and need variables based on the Andersen framework of health service use. We constructed a study cohort of HCBS recipients who received a standardized clinical assessment in 2022, which provided predictor data. Medicaid and Medicare claims data provided NH admission data for 12 months after the assessment date. Multinomial regression analysis compared predictors among non-NH users, short-term, and long-term users. The study cohort (n = 6,040) was 75.4% female, with mean(sd) age=80.4(8.9); 49.5% non-Hispanic white, 29.8% Hispanic, and 14.7% non-Hispanic Black individuals; 12.3% experienced short-term and 8.7% long-term NH admissions. Compared to non-NH users, short-term NH users were more likely to be > 85 years old (relative risk ratio (RRR)=1.51) have >6 comorbidities (RRR=1.76), and less likely to be Hispanic individuals (RRR=0.49; all p < 0.001). Long-term NH users were more likely to have dementia (RRR=1.33) and severe levels of ADL disability (RRR=2.0), and less likely to be Hispanic (RRR=0.29) or non-Hispanic Black individuals (RRR=0.68; all p < 0.01). Findings will enable improved targeting opportunities of HCBS recipients at greater risk for NH admission for policy makers and providers.

